# Indomethacin Increases the Efficacy of Oxygen Utilization of Colonic Mitochondria and Uncouples Hepatic Mitochondria in Tissue Homogenates From Healthy Rats

**DOI:** 10.3389/fmed.2020.00463

**Published:** 2020-08-21

**Authors:** Anna Herminghaus, Albert J. Buitenhuis, Jan Schulz, Richard Truse, Christian Vollmer, Borna Relja, Inge Bauer, Olaf Picker

**Affiliations:** ^1^Department of Anaesthesiology, University Hospital Duesseldorf, Duesseldorf, Germany; ^2^Experimental Radiology, Department of Radiology and Nuclear Medicine, Otto von Guericke University, Magdeburg, Germany

**Keywords:** indomethacin, mitochondrial function, colon, liver, adverse event

## Abstract

**Background:** Studies suggest that indomethacin (Indo) exhibits detrimental changes in the small intestine (microvascular disorder, villus shortening, and epithelial disruption), mainly due to mitochondrial uncoupling. The effects of Indo on colon and liver tissue are unclear. The aim of this study was to determine the effects of Indo on mitochondrial respiration in colonic and hepatic tissue.

**Methods:** Mitochondrial oxygen consumption was assessed in colon and liver homogenates from healthy rats. Homogenates were incubated without drug (control) or Indo (colon: 0.36, 1, 30, 179, 300, 1,000, 3,000 μM; liver: 0.36, 1, 3, 10, 30, 100, 179 μM; *n* = 6). State 2 (substrate-dependent) and state 3 (ADP-dependent respiration) were evaluated with respirometry. The respiratory control index (RCI) was derived and the ADP/O ratio was calculated.

**Statistics:** Data presented as % of control, min/median/max, Kruskal–Wallis+Dunn's correction, ^*^p < 0.05 vs. control.

**Results:** Indo had no effect on RCI of colonic mitochondria. ADP/O ratio increased in complex I at concentrations of 1,000 and 3,000 μM (Indo 1,000 μM: 113.9/158.9/166.9%^*^; Indo 3,000 μM: 151.5/183.0/361.5%^*^) and in complex II at concentrations of 179 and 3,000 μM vs. control (179 μM: 111.3/73.1/74.9%^*^; 3,000 μM: 132.4/175.0/339.4%^*^). In hepatic mitochondria RCI decreased at 179 μM for both complexes vs. control (complex I: 25.6/40.7/62.9%^*^, complex II: 57.0/73.1/74.9%^*^). The ADP/O ratio was only altered in complex I at a concentration of 179 μM Indo vs. control (Indo 179 μM: 589.9/993.7/1195.0 %^*^).

**Conclusion:** Indo affected parameters of mitochondrial function in an organ-specific and concentration-dependent manner. In colonic tissue, RCI remained unaltered whereas the ADP/O ratio increased. Indo at the highest concentration decreased the RCI for both complexes in hepatic mitochondria. The large increase in ADP/O ratio in complex I at the highest concentration likely reflects terminal uncoupling.

## Introduction

Non-steroidal anti-inflammatory drugs (NSAIDs) are widely used for various indications. They are generally considered as safe medication, but are also known to cause different adverse events, which potentially limit their use. The main complication of the therapy with NSAIDs is gastrointestinal (GI) damage. Although the most frequent disturbances are upper GI-bleeding, adverse events affecting lower GI still account for 40% of all NSAID-related serious GI complications (1). It is generally accepted that the inhibition of cyclooxygenase (COX), followed by multiple pathogenic events like increased intestinal permeability, infiltration of neutrophils and microcirculatory dysregulation play a critical role in the development of inflammation and ulcers (2, 3). Further studies suggest that mucosal concentration of prostaglandins could be decreased without mucosal damage and that inhibition of COX 1 and 2 causes gastrointestinal lesions, but less severe than those caused by NSAIDs (4, 5). Thus, inhibition of COX does not seem to be the single mechanism of NSAID-induced gastrointestinal damage. Evidence suggests that affecting mitochondrial function with a consecutive decrease in cellular ATP-production may be the underlying biochemical mechanism of the toxicity of NSAIDs (3). However, data available on effects of NSAIDs on mitochondrial function are inconsistent showing impaired (6) or unchanged (7) mitochondrial function in rat jejunum. Similar to upper gastrointestinal complications, the pathogenesis and prevention of NSAID-associated lower GI complications remain unclear (8). A number of studies suggest that NSAIDs can cause damage of the large intestine by a negative impact on the mucosal permeability (9). Data concerning mitochondrial function in the colon under NSAIDs-therapy are lacking completely.

NSAIDs are also associated with hepatotoxicity ranging from asymptomatic elevations in serum aminotransferase levels to severe liver failure (10, 11). Although clinically apparent liver injury from NSAIDs is rare (~1–10 cases per 100,000 prescriptions) the massive consumption of NSAIDs worldwide makes them an important cause of drug-induced liver injury (12). The molecular mechanisms underlying this toxicity have not yet been fully clarified. However, experimental data suggests that they include increased drug concentration in the hepatobiliary compartment, formation of reactive metabolites that cause oxidative stress, and mitochondrial dysfunction (13). In this context, the mitochondrial dysfunction is mainly described as an uncoupling and reduction of ATP production (6, 14, 15). Lipophilic and weakly acidic drugs like NSAIDs can easily pass through the outer mitochondrial membrane and shuttle protons from the intermembraneous space back into the matrix. This proton cycling scatters the proton gradient which is continuously maintained by the electron transport chain. This weakens the activity of the ATP synthetase, which is normally driven by this proton gradient (13). There is also ambiguity concerning toxic concentrations of NSAIDs affecting mitochondrial function. While an uncoupling effect of indomethacin on hepatic mitochondria could be shown with concentrations lower than 200 μM (14) another study rather suggests a stimulating effect of indomethacin on mitochondrial respiration at lower concentrations (0.02–2.7 μM) (16). The comparison of NSAID-induced cytotoxicity *in vitro* showed a large difference between compounds which was not related to their chemical structures (15). Indomethacin seems to be among the most cytotoxic NSAIDs and serves a gold standard to study NSAID toxicity (3, 17, 18).

Taken together, the effects of NSAIDs on mitochondrial function in different organs have been insufficiently examined. Data concerning hepatic mitochondria are inconsistent and are lacking completely for other organs like the colon. The aim of this study was therefore to investigate the concentration dependent effect of indomethacin on mitochondrial respiration in hepatic and colonic tissue homogenates from healthy rats.

## Materials and Methods

### Animals

The study was approved by the Animal Ethics Committee of the University of Duesseldorf, Germany (project identification code: O27/12) and conducted in accordance with the Guide for the Care and Use of Laboratory Animals of the National Institutes of Health (19).

Male Wistar rats (~3 months old) were kept at an artificial 12-h light/dark cycle at controlled room temperature with free access to standard chow and water. 48 rats were sacrificed by decapitation under deep sedation with sodium pentobarbital (90 mg/kg) and liver and colon tissues were harvested.

### Preparation of Liver and Colon Homogenates

Liver and colon homogenates were prepared as described previously (20–22). Briefly, liver tissue was placed in 4°C-cold isolation buffer (130 mM KCl, 5 mM K_2_HPO_4_, 20 mM MOPS, 2.5 mM EGTA, 1 μM Na_4_P_2_O_7_, 0.1% bovine serum albumin (BSA) pH 7.15), minced into 2–3-mm^3^ pieces, rinsed twice in isolation buffer to remove traces of blood, and homogenized (Potter-Elvehjem, 5 strokes, 2,000 rpm). Freshly harvested colon tissue was placed in 4°C-cold isolation buffer (as for the liver but containing 2% BSA), opened longitudinally, and cleaned softly with a cotton compress. After treatment with 0.05% trypsin for 5 min on ice, the tissue was placed in 4°C-cold isolation buffer containing 2% BSA and protease inhibitors (cOmplete™ Protease Inhibitor Cocktail, Roche Life Science, Mannheim, Germany), minced into 2–3-mm^3^ pieces, and homogenized (Potter-Elvehjem, 5 strokes, 2,000 rpm). Protein concentration in the tissue homogenates with supplemented BSA was determined using Lowry method (23) with bovine serum albumin as a standard. We used a higher BSA concentration for the preparation of colon homogenates than for liver homogenates to prevent an uncoupling effect of fatty acids, which are present in the submucosa of the colon (24).

### Measurement of Mitochondrial Respiratory Rates

The assessment of mitochondrial respiration was performed after addition of carrier substance (DMSO, Sigma Aldrich Chemie GmbH, Steinheim, Germany)—control, or different concentrations of indomethacin (Sigma Aldrich Chemie GmbH, Steinheim, Germany) (0.36, 1, 30, 179, 300, 1,000, and 3,000 for the colon and 0.36, 1, 3, 10, 30, 100, and 179 μM for the liver). The incubation took place at room temperature (kept at 21°C) for 3 min. Six biological and 2–3 technical (2–3 separate measurements from a single homogenate) replicates were performed.

Mitochondrial oxygen consumption was measured as described previously (21, 22). Briefly, the measurement was performed at 30°C using a Clark-type electrode (model 782, Strathkelvin instruments, Glasgow, Scotland). Tissue homogenates were suspended in respiration medium (130 mM KCl, 5 mM K_2_HPO_4_, 20 mM MOPS, 2.5 mM EGTA, 1 μM Na_4_P_2_O_7_, 0.1% BSA for liver, and 2% BSA for colon, pH 7.15) to yield a protein concentration of 4 or 6 mg/ml for liver and colon, respectively.

Mitochondrial state two respiration was measured in the presence of either substrates for complex I–glutamate and malate (both 2.5 mM, G-M) or for complex II–succinate (10 mM for liver, 5 mM for colon, S) combined with 0.5 μM rotenone–the inhibitor of complex I activity.

The maximal mitochondrial respiration (state 3) was recorded after the addition of ADP (250 μM for liver, 50 μM for colon). The concentrations of ADP were empirically adapted to the different tissue homogenates (hepatic and colonic tissue) in pilot experiments and as published previously (21, 22, 25). We used different concentrations of ADP for liver and colon since we identified these concentrations as non-saturated for the used amount of protein (4 mg/ml for the liver and 6 mg/ml for the colon). The respiratory control index (RCI) was calculated (state 3/state 2) to define the coupling between the electron transport system (ETS) and oxidative phosphorylation (OXPHOS). To reflect the efficacy of OXPHOS, the ADP/O ratio was calculated from the amount of ADP added and oxygen consumed in state 3. The average oxygen consumption was calculated as the mean from two or three technical replicates.

Respiration rates were expressed as nanomoles O_2_ per minute per milligram protein. Mitochondria were checked for damage by the addition of 2.5 μM cytochrome c and 0.05 μg/ml oligomycin. Lack of increase in flux after the addition of cytochrome c indicated integrity of the mitochondrial outer membrane. After inhibition of the ATP synthesis by oligomycin, the mitochondria were transferred to state 2, which reflects the respiration rate compensating the proton leak. The lack of difference in O_2_ consumption after adding oligomycin compared to state 2 indicates that the inner membrane was intact, and mitochondria were not damaged through the preparation procedure.

### Statistical Analysis

Statistical analysis was conducted using GraphPad Prism 8.0 (GraphPad Software, Inc, La Jolla, USA). After testing for normality (Kolmogorov-Smirnov) the non-parametric data were analyzed using the Kruskal-Wallis test of variance followed by *post-hoc* Dunn's correction. Data are presented as percentage of control values, *p* < 0.05 was considered significant.

## Results

### Effect of Indomethacin on Mitochondrial Respiration in Colon Homogenate

**Figure 1:**
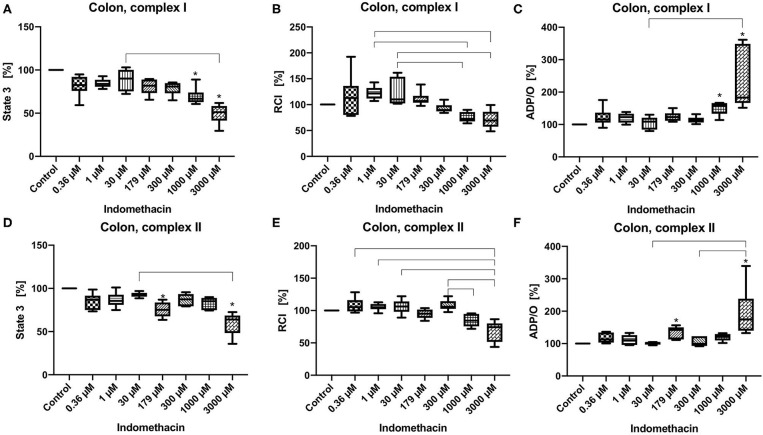
Effect of indomethacin (0.36, 1, 30, 179, 300, 1,000, and 3,000 μM) on colonic mitochondrial function: State 3 for complex I **(A)** and II **(D)**, respiratory control index (RCI) for complex I **(B)** und II **(E)** and ADP/O ratio for complex I **(C)** and II **(F)**. Data are shown as percentage of the control value (median/min/max), *n* = 6, *p < 0.05 vs. control, 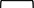
*p* < 0.05 between groups.

State 3 was reduced at concentrations of 1,000 and 3,000 μM for complex I (control 100%, 1,000 μM: 60.63/66.66/88.9%; 3,000 μM: 29.56/51.23/61.75%) (Figure 1A) and at 179 μM and 3,000 μM for complex II (control 100%, 179 μM: 63.53/75.15/86.93%; 3,000 μM: 35.87/63.97/72.61%) (Figure 1D). The RCI remained unchanged for both complexes under the influence of indomethacin (Figures 1B,E). An increase in ADP/O ratio was seen at 1,000 and 3,000 μM for complex I (control 100%, 1,000 μM: 113.9/158.9/166.9%; 3,000 μM: 151.5/183.0/361.5%) (Figure 1C) and at 179 and 3,000 μM for complex II (control 100%, 179 μM: 111.3/142.2/156.7%; 3,000 μM: 132.4/175.0/339.4%) (Figure 1F). Original data on State 2, 3, and 4 are presented in Supplementary Material.

### Effect of Indomethacin on Hepatic Mitochondrial Function

**Figure 2:**
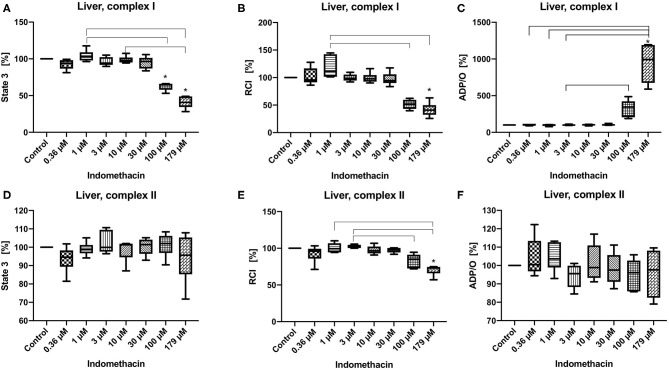
Effect of indomethacin (0.36, 1, 3, 10, 30, 100, and 179 μM) on hepatic mitochondrial function: State 3 for complex I **(A)** and II **(D)**, respiratory control index (RCI) for complex I **(B)** und II **(E)** and ADP/O ratio for complex I **(C)** and II **(F)**. Data are presented as percentage of the control value (median/min/max), *n* = 6, *p < 0.05 vs. control, 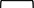
*p* < 0.05 between groups.

State 3 was decreased only for complex I at 100 and 179 μM (control 100%, 100 μM: 53.08/64.63/66.19%; 179 μM: 28.14/40.65/49:00%) (Figure 2A). The RCI was decreased for both complexes but only at the highest concentration of 179 μM (control 100%, complex I: 28.14/40.65/49.00%, complex II: 57.01/73.11/74.88%) (Figures 2B,E). The ADP/O ratio was increased only for complex I at 179 μM (control 100%, 179 μM: 589.9/993.7/1195.00%) (Figure 2C). State 3 and the ADP/O ratio for complex II stayed unchanged (Figures 2D,F). Original data on State 2, 3 and 4 are presented in Supplementary Material.

## Discussion

The main results from this study are that indomethacin increases the efficacy of oxygen utilization of colonic mitochondria but uncouples hepatic mitochondria, predominantly through complex I.

The experimental setting is well-established and based on our previous publications (21, 22). The chosen concentrations of indomethacin are based on data from similar *in vitro* studies with isolated hepatic mitochondria and cell culture (14, 17, 26). The used concentrations of the drug differ between the tissues. We adjusted the drug concentrations since the maximal uncoupling of the respiratory chain from the ATP-synthase in hepatic mitochondria was observed after stimulation through complex I at a concentration of 179 μM. Since in contrast, in colonic mitochondria indomethacin hardly showed any effect up to 179 μM, we additionally applied higher drug concentrations.

The applied drug concentrations include the clinically relevant range as well as higher concentrations. After oral administration of an average dose of 75–200 mg/day the plasma concentrations in humans (27) reach 0.3–3 μM and after i.v., application of 0.2 mg/kg in sheep (28) reach 14 μM, however with a high inter-individual variability–up to 20 fold (29). In rats, the plasma concentration after an oral dose of 10 mg/kg reached 140 μM (30). Generally, indomethacin is dosed higher (10–40 mg/kg) for rats (6, 7, 30) than for humans (1 mg/kg) (27). Thus, our results may be relevant for the *in vivo* situation since adverse events already occur with recommended dosages and aggravate after accidental or intentional overdosing of the drug (31). Moreover, many patients suffer from multimorbidity. Preexisting or NSAIDs-induced renal and/or liver insufficiency can also result in supraclinical or even toxic drug plasma concentrations (31, 32).

In colonic mitochondria indomethacin at higher concentrations (1,000 and 3,000 μM for complex I and 179 and 3,000 μM for complex II) reduced state 3 without affecting the RCI and increased ADP/O ratio. As the RCI reflects the coupling between the respiratory chain and the ATP-synthase and the ADP/O-ratio reflects the efficacy of the oxidative phosphorylation, indomethacin seems to have rather positive effects on colonic cell metabolism resulting in a more effective ATP-production. Based on our data, we cannot explain the lack of effect of indomethacin at 300 and 1,000 μM on colonic mitochondria after stimulation of the respiratory chain through complex II. To address this question more deeply, further investigations, like assessment of the activities of the single complexes are necessary. To the best of our knowledge, we are the first to examine the effect of indomethacin on colonic mitochondrial function. Jacob et al. investigated the impact of this drug on jejunal mitochondria. In their study, no alterations were observed in the mitochondrial oxygen uptake, neither *ex vivo*, nor *in vitro* (7). The difference between their and our results could be explained by the different tissues investigated (jejunum vs. colon) and different experimental settings (whole tissue vs. tissue homogenate). However, the drug concentrations used in the study by Jacob et al. were similar to the concentrations, which caused an alteration of oxygen uptake in our experiments (2.5 vs. 1–3 mM). Basivireddy et al. examined the influence of indomethacin on mitochondrial function in enterocytes isolated from rat small intestine and obtained different results compared to those of Jacob et al. and to ours (6). In their experiments rats were dosed with 40 mg/kg indomethacin by gavage. They showed a decreased RCI, swelling of the mitochondria and a decreased influx of calcium into the mitochondria. These changes could be interpreted as rather harmful to the cell. Also, in this case the differences could be explained by the different tissues, divergent application forms (oral application in *in vivo* experiments vs. *in vitro* incubation) and possibly varying drug concentrations. Since the plasma concentrations of indomethacin after oral application are well-examined, our knowledge about tissue concentrations of the drug is very limited. There are only few data about calculated hepatic concentration of indomethacin (33) and data about intestinal concentrations are lacking completely.

The effect of indomethacin on hepatic mitochondria was different than on colonic mitochondria. We observed a dose-dependent and complex-specific effect. The impact of indomethacin on complex II was mild—only the high concentration (179 μM) decreased RCI without affecting state 3 and ADP/O ratio. When the respiratory chain was stimulated through complex I, indomethacin at higher concentrations (100 and 179 μM) decreased state 3 and consecutively the RCI (but only at a concentration of 179 μM). The dramatically reduced oxygen consumption during state 3 led to an increase of ADP/O ratio (ADP added/oxygen consumed), which might be interpreted as toxic discoupling rather than an increase of the efficacy of the oxidative phosphorylation. A selective or stronger effect of a drug on one particular complex of the respiratory chain is a well-known phenomenon. For instance, metformin is known to inhibit complex I selectively (34). Furthermore, antipsychotic drugs like haloperidol, and to a lesser degree chlorpromazine and risperidone have been found to inhibit only complex I. Antidepressant drugs are able to interact with complexes I, II, and IV, but the inhibition of complex I is most pronounced (35). It is not fully elucidated, why some drugs have stronger affinity to certain complexes. It is suggested that the lipophilic domain of the antidepressant drugs can compete with the substrate for binding to complex I. Concerning indomethacin, Boushel et al. suggested in their study on human muscle mitochondria that the underlying mechanisms of stronger inhibition of complex I include leakage of electrons, iron release and superoxide and hydroxyl radical production (36). Moreover, the drug effect can be organ specific, like by serotonin reuptake inhibitors, which block complex I and IV in the brain, but complex I and V in the liver (35).

Our results concerning hepatic mitochondria are not in line with the results of other researchers. Somasundaram et al. could show a dose dependent effect of indomethacin on hepatic mitochondrial function for complex II. In their experiments, stimulation of respiration (state 3) occurred at concentrations up to 0.5 mM, followed by a progressive decrease in oxygen consumption with further increases in drug concentrations up to 2 mM, which is indicative of inhibition of the electron transport chain (26). The effect of indomethacin on the mitochondrial function after stimulation of the respiratory chain through complex I was not examined. Mahmud et al. also tested the influence of indomethacin on hepatic mitochondria but again only for complex II. They achieved similar results as Somasundaram et al. however, at substantially lower concentrations of the drug. They observed a stimulating effect on state 3 respiration up to 0.5 μM and inhibitory effect at higher concentrations (up to 3 μM) (16).

The effect of indomethacin on hepatic mitochondria is similar to the effects of other NSAIDs. Most NSAIDs are lipid soluble weak acids, which is a common feature of all uncouplers of oxidative phosphorylation (37). Acetylsalicylic acid, naproxen, and piroxicam show a similar uncoupling effect on hepatic mitochondria to that of indomethacin (14, 26). Moreover, some of the NSAIDs like diclofenac or fenoprofen reduce the ATP concentration in isolated hepatocytes (15). Data about NSAIDs and colon are lacking in the literature.

Our results showing opposite effects of the drug on mitochondrial function in different tissues confirming the well-accepted theory that the impact of various stimuli on mitochondria is organ specific (38).

## Conclusions

Taken together, indomethacin has organ-specific, dose-dependent, and complex-specific effects on mitochondrial respiration. While it shows rather positive effects on colonic mitochondrial function, the impact on oxidative phosphorylation in the liver is rather deleterious.

This could possibly be one of the mechanisms contributing to indomethacin-related hepatotoxicity. However, this hypothesis must be considered very carefully. The transfer of results performed *in vitro* using tissues from young and healthy animals into human medicine can only be performed with reservation. Moreover, adverse effects are complex processes including many factors like pre-existing organ damage and co-medication.

The positive effect of indomethacin on colonic mitochondrial respiration cannot explain the lower GI-complications described under NSAID therapy. To better understand the pathomechanisms of these adverse events, further research on this field is needed. Our data extend our knowledge about a possible mode of action of NSAIDs and offer a new insight into conceivable mechanisms of their side effects.

## Data Availability Statement

All datasets generated for this study are included in the article/Supplementary Material.

## Ethics Statement

The animal study was reviewed and approved by University of Duesseldorf, Universitaetstrasse 1, 40225 Duesseldorf, Germany.

## Author Contributions

AH, IB, and OP: conceptualization, supervision, and project administration. AH and AB: methodology, software, investigation, and data curation. AH, AB, JS, BR, and RT: validation. AH, AB, IB, and OP: formal analysis. IB and OP: resources. AH: writing—original draft preparation and visualization. AH, AB, JS, RT, BR, CV, IB, and OP: writing—review and editing. All authors contributed to the article and approved the submitted version.

## Conflict of Interest

The authors declare that the research was conducted in the absence of any commercial or financial relationships that could be construed as a potential conflict of interest.
