# Supplementary material for: Indomethacin Increases the Efficacy of Oxygen Utilization of Colonic Mitochondria and Uncouples Hepatic Mitochondria in Tissue Homogenates From Healthy Rats
Source: Front Med (Lausanne). 2020 Aug 21;7:463. doi: 10.3389/fmed.2020.00463 (PMC7472952; doi:10.3389/fmed.2020.00463)
Supplement: Supplementary file 1 [file Table_1.DOCX]

| Liver | | | | | | |
| --- | --- | --- | --- | --- | --- | --- |
|  | State 2 [nmol/mg/min] | | State 3 [nmol/mg/min] | | State 4 [nmol/mg/min] | |
|  | Complex I | Complex II | Complex I | Complex II | Complex I | Complex II |
| [µM] | MW±SD | MW±SD | MW±SD | MW±SD | MW±SD | MW±SD |
| Contr. | 1.82±0.55 | 2.7±0.88 | 12.16±3.31 | 17.13±5.02 | 5.02±1.29 | 7.28±2.07 |
| 0.36 | 1.52±0.54 | 2.16±0.44 | 9.31±1.11 | 13.4±2.17 | 6.0±2.11 | 7.15±1.47 |
| 1 | 1.6±0.41 | 2.63±0.71 | 11.44±4.07 | 14.83±5.42 | 7.46±2.84 | 9.41±4.46 |
| 3 | 1.42±0.27  | 2.35±0.34 | 10.72±1.93 | 15.68±2.55 | 4.27±0.4 | 6.54±0.5 |
| 10 | 1.74±0.2 | 2.48±0.22 | 11.59±2.42 | 16.18±3.43 | 4.9±1.14 | 7.14±1.5 |
| 30 | 1.67±0.21 | 2.62±0.44 | 12.28±1.56 | 18.07±2.98 | 4.22±0.99 | 6.55±0.87 |
| 100 | 2.01±0.63 | 3.1±0.93 | 7.87±2.07 | 18.07±5.42 | 7.17±1.93 | 7.57±1.94 |
| 179 | 2.85±2.19 | 5.48±4.04 | 6.16±2.09 | 20.07±7.07 | 5.24±1.76 | 10.06±3.44 |

Table 1. State 2, 3 and 4 for complex I and II in liver homogenate.

| Colon | | | | | | |
| --- | --- | --- | --- | --- | --- | --- |
|  | State 2 [nmol/mg/min] | | State 3 [nmol/mg/min] | | State 4 [nmol/mg/min] | |
|  | Complex I | Complex II | Complex I | Complex II | Complex I | Complex II |
| [µM] | MW±SD | MW±SD | MW±SD | MW±SD | MW±SD | MW±SD |
| Contr. | 0.65±0.24 | 0.76±0.28 | 1.44±0.46 | 2.41±0.72 | 0.77±0.34 | 1.36±0.48 |
| 0.36 | 0.44±0.05 | 0.46±0.11 | 0.91±0.25 | 1.25±0.25 | 0.39±0.18 | 0.69±0.25 |
| 1 | 0.46±0.1 | 0.53±0.09 | 1.16±0.26 | 1.71±0.42 | 0.55 ±0.21 | 0.88±0.2 |
| 30 | 0.47±0.08  | 0.64±0.13 | 1.22±0.28 | 2.21±0.46 | 0.67±0.17 | 1.32±0.26 |
| 179 | 0.88±0.55 | 1.07±0.9 | 1.32±0.69 | 2.0±1.21 | 0.73±0.49 | 1.22±0.84 |
| 300 | 0.4±0.07 | 0.5±0.16 | 0.96±0.32 | 1.88±0.8 | 0.61±0.34 | 1.24±0.58 |
| 1000 | 0.48±0.12 | 0.63±0.12 | 1.1±0.33 | 2.15±0.63 | 0.69±0.33 | 1.25±0.43 |
| 3000 | 0.41±0.07 | 0.66±0.12 | 0.94±0.23 | 1.91±0.64 | 0.51±0.17 | 1.11±0.28 |

Table 2. State 2, 3 and 4 for complex I and II in colon homogenate.
